# The Necessity for Trained and Educated Health Officials

**Published:** 1893-09

**Authors:** 


### The Necessity for Trained and Educated Health Officials.

There has probably been no time in the history of this country when trained, competent and efficient health officers were so much needed as they are now. The average health officer is appointed without any special training or qualification for the place; his tenure of office is so slight that very few feel warranted in qualifying themselves for such duties and in no other direction is there any special incentive to full preparation for the discharge of the responsibilities that devolve upon the holder of such office. Much has recently been said with regard to the manner in which cholera was managed in England during the last autumn, but nothing was said in regard to the special knowledge required of medical officers of health in that country, before they receive such appointments. No one is eligible to such appointment in Great Britain without special training and his qualification having been established by examination; his position is then assured and he is not subject to removal with change of administration.

It ought to be axiomatic that no health officer, no health commissioners, no executive officer of a board of health, should anywhere be appointed, until after thorough examination, or training in a subordinate capacity, and until he had evinced special aptitude for and interest in that line of work. A great misfortune in this country is that whenever a change of administration is brought about, it is considered necessary to change officers of health and to appoint a new man without any reference to his special capability. Instances frequently occur, involving great responsibility, where the appointee is wholly incompetent, and is dependent upon the subordinates in his office for information and advice; in short, is compelled to learn the duties incumbent upon him at the expense of the life and treasure of the public. Until a change is made in the mode of seleotion of these officers, and the tenure by which they hold their appointments is more stable, we cannot expect in this country efficient sanitary administration. —Ed. *Am. Med. Ass'n. Journal. Feb.* 18, 1893.

